# Acute Appendicitis and Small Bowel Obstruction Secondary to Metastatic Breast Cancer

**DOI:** 10.7759/cureus.4706

**Published:** 2019-05-21

**Authors:** Laith Numan, Samia Asif, Omar K Abughanimeh

**Affiliations:** 1 Internal Medicine, University of Missouri-Kansas City School of Medicine, Kansas City, USA; 2 Hematology/Oncology, University of Nebraska Medical Center, Omaha, USA

**Keywords:** acute appendicitis, invasive lobular breast cancer, gastrointestinal metastasis, small bowel obstruction

## Abstract

Breast cancer is the most common cancer in women; it is well-known to metastasize to lymph nodes, lungs, liver, brain, and bones. However, luminal gastrointestinal metastasis is rare, especially to the appendix. Herein, we report a case where cancer metastasized to the ileum and appendix, causing acute appendicitis and small bowel obstruction. This is a 44-year-old female with a history of stage IV metastatic breast cancer to bones, lungs, and ovaries, presented with acute abdominal pain for one day. Her abdomen was soft, distended with generalized tenderness. Computed tomography (CT) of the abdomen and pelvis showed a partial small bowel obstruction and swollen appendix. After her symptoms worsened on conservative treatment, she was taken to the operating room where she was found to have a markedly dilated ileum with signs of acute appendicitis, so she underwent ileocecectomy, appendectomy, and lysis of adhesions. Pathology showed metastatic breast cancer in the appendix with findings consistent with acute appendicitis. She tolerated surgery well without complications. In conclusion, small intestinal and appendiceal metastases of breast cancer are very rare though they should be considered in the differential diagnosis in cancer patients presenting with acute abdominal pain.

## Introduction

Breast cancer is currently the most common type of malignancy [1], with an observed incidence rate of 127.5 per 100, 000 women per year according to the Surveillance, Epidemiology and End Results (SEER) program [2]. Patients with distant metastases have a five-year relative survival rate of 27.4% (2009-2015) [2]. The most common sites of metastases of primary breast cancer include regional lymph nodes, bones, liver, lung, brain, and skin [3]. However, luminal gastrointestinal (GI) metastasis is rare, especially to the appendix. Literature review reveals only 15 prior cases of metastatic breast cancer (MBC) with the involvement of the appendix, with most patients undergoing appendectomy [4-5]. Since this is a rare event, which may cause acute appendicitis and carries a high risk of perforation, early recognition, and appropriate management is crucial. We present the case of a 44-year-old lady with estrogen receptor (ER) and progesterone receptor (PR) positive, human epidermal growth factor receptor 2 (HER-2) negative MBC who was evaluated for abdominal pain and subsequently diagnosed with acute appendicitis secondary to appendiceal metastases.

## Case presentation

A 44-year-old female with a past medical history of stage IV breast cancer with metastasis to the bones, lungs, and ovaries. Her breast cancer was diagnosed three years before presentation. She was diagnosed with right invasive lobular carcinoma (ER-positive, PR-positive, and HER-2 negative), she had a right mastectomy and was started on tamoxifen. One year later, she had metastasis to the left breast and lungs, so she was started on exemestane and everolimus with stability in disease till her current presentation.

**Figure 1:**
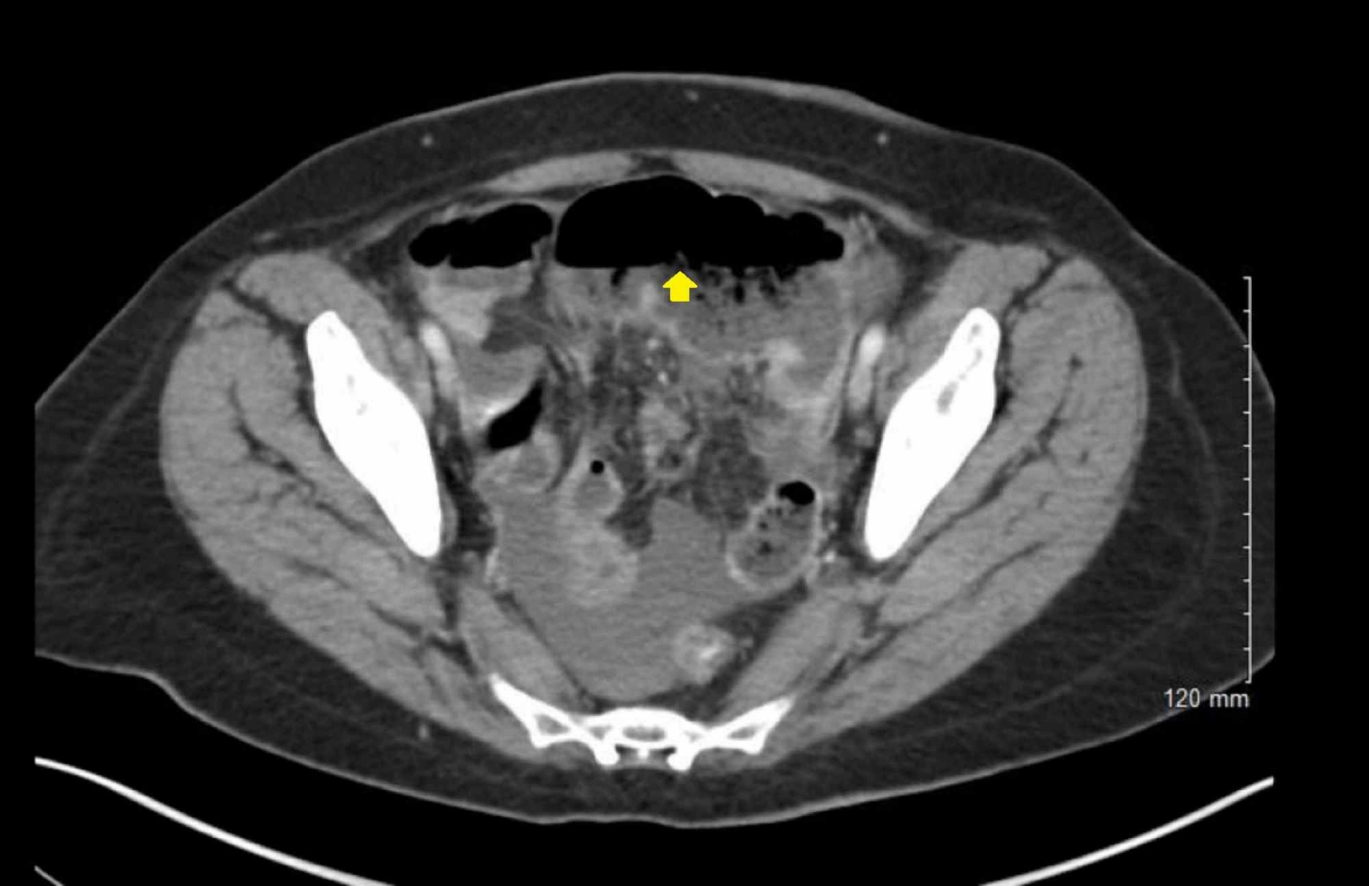
A CT scan of the abdomen (cross section) showing a fluid-filled loop of the small bowel suspicious for SBO. CT: Computed Tomography; SBO: Small Bowel Obstruction.

**Figure 2:**
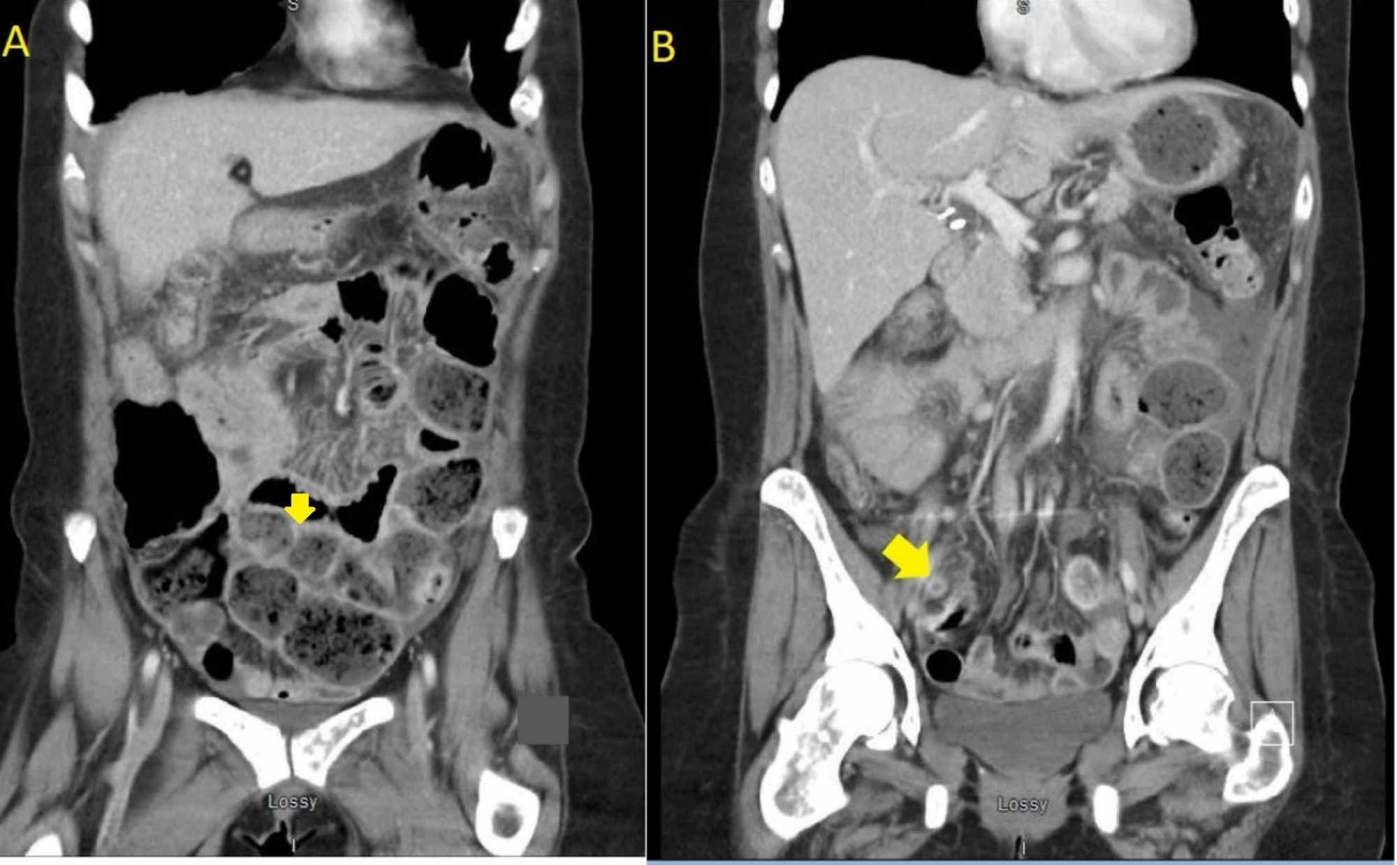
Coronal sections of the CT scan of the abdomen and pelvis. A: Showing dilated bowel lobes suggesting intestinal obstruction; B: Arrow pointing at the swollen appendix. CT: Computed Tomography

The patient presented to the emergency department with acute abdominal pain of one-day duration. Her pain was generalized mostly in the lower abdomen but not localized to any specific area. The pain was associated with nausea, vomiting, abdominal distention, and constipation. On abdominal examination, her abdomen was soft and distended, with generalized tenderness. X-ray of the abdomen showed signs of obstruction with dilated bowel loops. Due to the severity of the pain, computed tomography (CT) of the abdomen and pelvis showed partial small bowel obstruction and a swollen appendix but did not meet the diagnostic criteria of acute appendicitis (Figures 1-2).

Her symptoms worsened despite conservative treatment, so she was taken to the operating room for exploratory laparotomy where she was found to have a markedly dilated ileum with peritoneal implants. Further exploration revealed an inflamed and edematous appendix surrounded by a foul-smelling fluid collection, so she underwent ileocecectomy, appendectomy, and lysis of adhesions. Pathology showed metastatic breast cancer in the mesentery, appendix, and small intestines with histological findings consistent of acute appendicitis. Her postoperative course was uncomplicated, and she was discharged home with follow-up with surgery and oncology.

## Discussion

Metastatic patterns of breast carcinoma have been studied, suggesting two important factors that impact the site of tumor spread: the estrogen receptor (ER) status and the pathology of cancer (ductal versus lobular) [6]. ER-positive cancer has a higher propensity to metastasize to bone whereas ER-negative tumors have a higher likelihood of visceral metastases. Similarly, lobular carcinoma metastasizes more to the gastrointestinal (GI) and gynecologic systems as well as to the peritoneum and the retroperitoneum. On the contrary, ductal carcinoma more often spreads to the liver, lungs, and brain [6]. Invasive lobular carcinoma accounts for 14% of all breast cancer cases [7].

Oldfield reported the first case of MBC manifesting as acute appendicitis in 1946 [8]. A study by Asch et al. reported, based on autopsy results from 337 patients with breast cancer, that 16.4% patients had GI metastases, but no cases of involvement of the appendix were seen [9].

The pathophysiology involves metastatic deposits to the serosa of the appendix progressing to encroach the lumen, leading to luminal obstruction. This predisposes to acute appendiceal inflammation and perforation. Microscopically, metastatic tumor cells are seen abundantly in the submucosa, muscularis mucosa, and subserosa, leading to luminal constriction. Distal to the site of obstruction by these tumor implants, edema of the appendiceal wall and infiltration by polymorphonuclear leukocytes is seen [10].

Symptoms may include nausea, vomiting, anorexia, and right lower abdominal pain. Physical examination may reveal fever and localized right lower quadrant tenderness. Appendix perforation and abscess formation may occur if there was any delay in treatment. Patients may or may not have leukocytosis. CT abdomen and pelvis is the preferred imaging modality. Imaging features of acute appendicitis on CT abdomen/pelvis include increased appendiceal wall thickening, peri-appendiceal fat stranding, appendiceal wall enhancement, and a calcified appendicolith in up to 30% of the patients [11]. Ultrasound and magnetic resonance imaging (MRI) may be performed if radiation exposure has to be avoided such as in pregnancy.

In a study by Connor et al., 7970 appendectomies performed over a period of 16 years were retrospectively reviewed; of these, only 74 patients, i.e., 0.9% of the patients had appendiceal tumors, out of which 12 were benign, 42 were carcinoids, and 20 were malignant; 49% had presented with acute appendicitis, which was the most common clinical presentation. Only 11 patients had a secondary malignancy involving the appendix; 55% of these were patients with primary colorectal cancer and appendiceal tumor involvement; the rest were reported as adenocarcinomas. Management varied from appendectomy to right hemicolectomy [12].

According to a comprehensive literature review by Ng et al., of the 15 known cases of MBC with appendiceal spread, seven had associated perforation. In terms of histopathology, 10 of the 15 cases had ductal and four had lobular pathology; three were of unknown type, and one was undifferentiated. Hormone receptor status was available for only four of these 15 cases (ER-positive in three, PR-positive in three, and HER-2 positive in two cases). Nine patients were treated with appendectomy alone while the remaining six required right hemicolectomy [13-14].

It is known that when appendicitis occurs in the setting of malignant metastases, these patients are immune compromised by advanced cancer itself as well as by chemotherapy, leading to late presentation and a higher risk of perforation. Also, chemotherapy side-effects, such as nausea, vomiting, and abdominal pain, may mimic signs and symptoms of acute appendicitis, resulting in delayed diagnosis [15]. Given the risk of increased morbidity and mortality, it remains essential to highlight this rare presentation of stage IV breast carcinoma so that physicians appropriately suspect this as the culprit lesion in the appropriate clinical setting. Given the high incidence of perforation in these patients, a prophylactic appendectomy can be offered to patients who will be requiring invasive intervention, such as oophorectomy or any other abdominal surgery [16], but there are no definitive guidelines in place yet.

This case was presented as a poster at the American College of Gastroenterology annual meeting (Abughanimeh O, Numan L, et al. Acute appendicitis and small bowel obstruction secondary to metastatic breast cancer. 2018. Program No. P0792. ACG 2018 Annual Scientific Meeting Abstracts. Philadelphia, Pennsylvania; American College of Gastroenterology).

## Conclusions

Small bowel obstruction along with acute appendicitis secondary to metastasis should be considered when forming the differential diagnosis for a patient presenting with severe abdominal pain and having a history of metastatic breast cancer, especially in invasive lobular carcinoma.
